# Examining Temporal Sample Scale and Model Choice with Spatial Capture-Recapture Models in the Common Leopard *Panthera pardus*

**DOI:** 10.1371/journal.pone.0140757

**Published:** 2015-11-04

**Authors:** Joshua F. Goldberg, Tshering Tempa, Nawang Norbu, Mark Hebblewhite, L. Scott Mills, Tshewang R. Wangchuk, Paul Lukacs

**Affiliations:** 1 Wildlife Biology Program, Department of Ecosystem and Conservation Science, College of Forestry and Conservation, University of Montana, Missoula, Montana, United States of America; 2 Ugyen Wangchuck Institute for Conservation and Environment, Department of Forests and Park Services, Ministry of Agriculture and Forests, Lamai Goempa, Bumthang, Bhutan; 3 Department of Forestry and Environmental Resources, North Carolina State University, Raleigh, North Carolina, United States of America; 4 Bhutan Foundation, Washington, D.C., United States of America; University of Zurich, SWITZERLAND

## Abstract

Many large carnivores occupy a wide geographic distribution, and face threats from habitat loss and fragmentation, poaching, prey depletion, and human wildlife-conflicts. Conservation requires robust techniques for estimating population densities and trends, but the elusive nature and low densities of many large carnivores make them difficult to detect. Spatial capture-recapture (SCR) models provide a means for handling imperfect detectability, while linking population estimates to individual movement patterns to provide more accurate estimates than standard approaches. Within this framework, we investigate the effect of different sample interval lengths on density estimates, using simulations and a common leopard (*Panthera pardus*) model system. We apply Bayesian SCR methods to 89 simulated datasets and camera-trapping data from 22 leopards captured 82 times during winter 2010–2011 in Royal Manas National Park, Bhutan. We show that sample interval length from daily, weekly, monthly or quarterly periods did not appreciably affect median abundance or density, but did influence precision. We observed the largest gains in precision when moving from quarterly to shorter intervals. We therefore recommend daily sampling intervals for monitoring rare or elusive species where practicable, but note that monthly or quarterly sample periods can have similar informative value. We further develop a novel application of Bayes factors to select models where multiple ecological factors are integrated into density estimation. Our simulations demonstrate that these methods can help identify the “true” explanatory mechanisms underlying the data. Using this method, we found strong evidence for sex-specific movement distributions in leopards, suggesting that sexual patterns of space-use influence density. This model estimated a density of 10.0 leopards/100 km^2^ (95% credibility interval: 6.25–15.93), comparable to contemporary estimates in Asia. These SCR methods provide a guide to monitor and observe the effect of management interventions on leopards and other species of conservation interest.

## Introduction

Across the globe, large carnivores face threats from habitat loss and fragmentation, prey depletion, and poaching [1]. These risk factors present an important conservation challenge because large carnivores can play critical ecological roles as keystone and umbrella conservation species [1–5]. The combination of the ecological impact of carnivores and risks to their persistence merit increased vigilance of their population status and trends to support effective management [6, 7]. However, many of these species challenge conventional monitoring methods, as large carnivores often occur at low densities in dense cover habitats, and have a shy, solitary, elusive, cryptic and/or nocturnal nature [8–12]. In short, large carnivores are frequently difficult to detect.

To address the imperfect detectability of large carnivores, researchers have adopted non-invasive, remote sampling techniques, such as automatically triggered camera traps, hair snares or scat surveys that likely reduce animal avoidance of target sites. For many species, these data can identify unique individuals based on genetics or unique markings, facilitating a capture-mark-recapture (CMR) analysis that explicitly incorporates detection probability into the estimate of abundance and/or density. Spatial capture-recapture (SCR) models further refine these techniques, by incorporating the spatial distribution of an individual’s movement inferred from observed captures [13, 14], which may be particularly appropriate given the territorial habits of many large carnivores. Furthermore, SCR models address a critical weakness within conventional CMR methods by linking estimates of abundance to a well-defined area, eliminating the need to estimate density with an ad-hoc estimate of area [14–17]. Simulations confirm that SCR analysis reduces bias in estimates of abundance or density under a range of conditions [12, 14, 18–20]. Additionally, SCR models can integrate ecological and behavioral factors into density estimates using individual, trap or spatial covariates, which can further improve the accuracy of estimates [12, 20–22].

Despite these advances, SCR methods raise new questions about sampling design and its relationship to population estimates and precision, particularly as these techniques are applied to new species and systems, including large carnivores. Russell *et al*. [23], Sollman *et al*. [18] and Sun *et al*. [19] examine model sensitivity to the spatial configuration of SCR study design. Wilton *et al*. [20] consider the arrangement of traps with reference to black bears, while Tobler and Powell [12] address issues of camera spacing and sampling area using parameter values specific to jaguars. Potential differences related to temporal variation in detections within SCR models remain untested, and guidelines developed for conventional CMR analysis may create confusion for practitioners of SCR methods. Conventional guidelines advise collapsing data temporally to improve detection probability [24, 25], despite the risk of lost detections and bias, and the potential for continuous-time analyses in an SCR framework [12, 26]. While these continuous-time models may provide the most technically sound approach, their recent development and complexity may limit their application in many conservation settings. Thus, understanding the relationship between sampling interval duration and the precision of density estimates in discrete-time models still hold practical value in maximizing the effectiveness and applicability of these approaches in a management setting.

Beyond sampling structure, the ability to incorporate covariates into density estimates raises important questions about model selection, particularly when estimates disagree. While maximum likelihood-based SCR estimation techniques have adopted an Akaike information criterion (AIC) model selection framework, no robust methods have been widely applied in Bayesian approaches to SCR analysis. These limitations in assessing the relative merits of different models restrict the usefulness of these methods, especially for data-poor species like large carnivores.

Here, we develop two methodological advances that may improve sampling design and analysis of capture-recapture studies for a variety of taxa: (1) we examine the impact of the temporal resolution of the data on SCR model estimates and precision and (2) we implement a Bayes factor approach to model selection with multiple covariates. We address these issues through a simulation study and using a remote camera-trapping data set for the common leopard (*Panthera pardus*) from the lower, subtropical foothills of Royal Manas National Park (RMNP), Bhutan. The common leopard occurs from sub-Saharan Africa to the Russian Far East, as well as on the islands of Sri Lanka and Java [27, 28] and faces threats similar to many other large carnivores. In Bhutan, human-wildlife conflict over livestock depredation has resulted in large numbers of leopards being killed [29–31]. Despite this threat to the common leopard population, knowledge of leopard populations is limited in spatial extent and relies on conventional CMR techniques [32]. Our application within this system and our simulations show that different sample interval lengths have a substantial impact on the precision of SCR density estimates and that Bayes factors can help discriminate between models including sex-specific capture probabilities and sex-specific distributions of movement. Given these results, we make recommendations for other capture-recapture studies, particularly those of large carnivores.

## Materials and Methods

### Study Area

**Fig 1:**
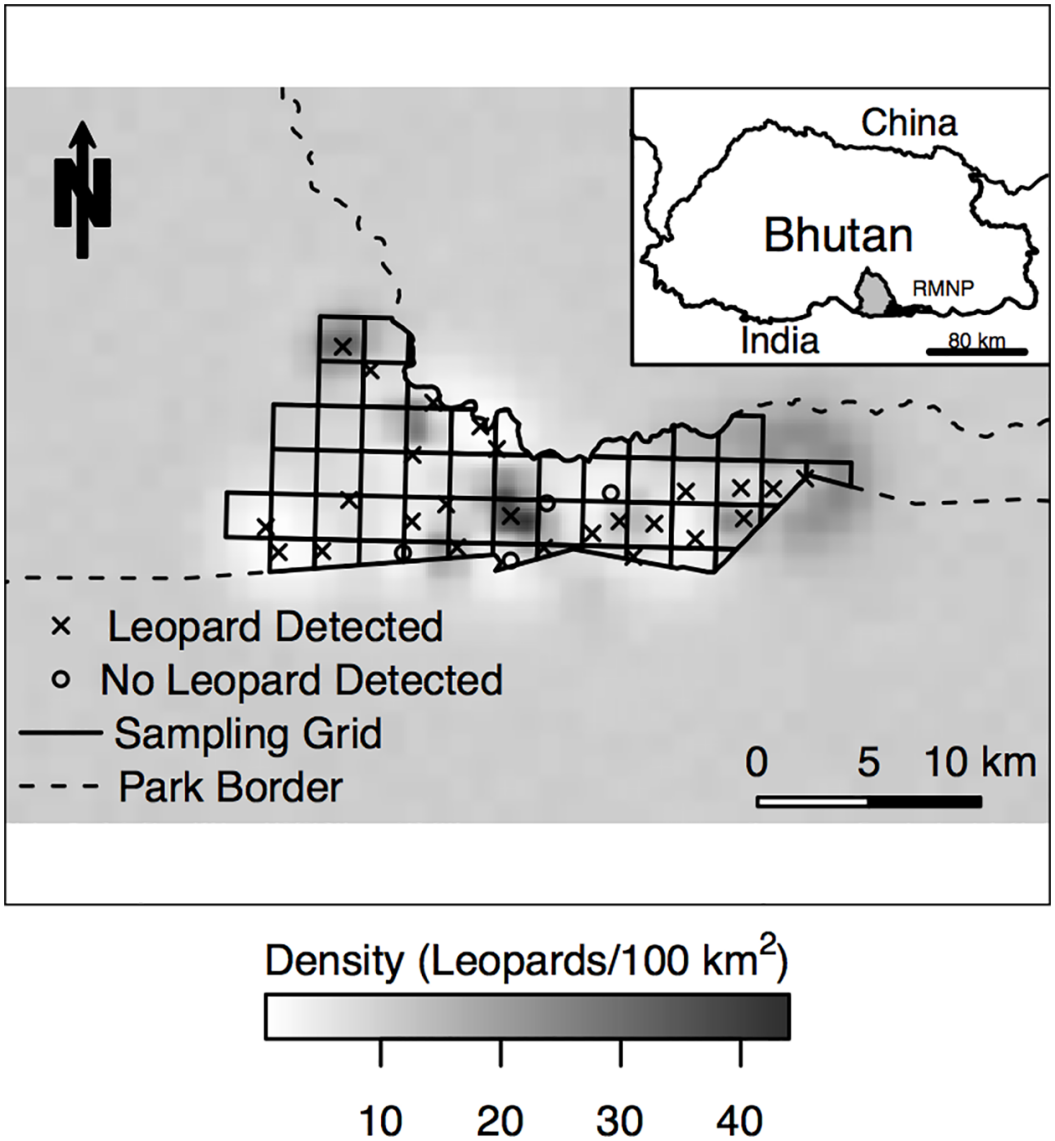
Spatial Density Estimate of Common Leopards *(Panthera pardus*) from the Best-Supported Model with Inset Study Area Map. The posterior spatial density estimate of common leopards/100 km^2^ from the best-supported spatial capture-recapture model, σ_sex_, in the lower foothills of Royal Manas National Park (RMNP), Bhutan for sampling carried out during 2010–2011. The 162 km^2^ sampling area is displayed with the solid black line, the RMNP boundary with broken black line, camera-trapping stations with leopard detections with crosses and camera-trapping stations without leopard detections black circles. Each station represents a pair of cameras. *Inset*: RMNP (light gray) in Bhutan with the location of the 162 km^2^ gridded study area (black) for common leopards in 2010–2011.

RMNP is located in the southern foothills of Bhutan (90^**°**^ 57’37.61” E, 26^**°**^ 47’31.27” N) and borders India’s Manas Tiger Reserve, forming a trans-boundary conservation landscape. RMNP covers 1057 km^2^, with elevations from 90 m in the southern foothills to 2900 m in the north (Fig 1). RMNP experiences hot, humid summers followed by cool, dry winters with annual maximum temperatures ranging from 20°C to 34°C. RMNP has diverse vegetation communities including; cool, moist and warm broadleaf forests, wetlands, subtropical dry forests, and subtropical scrub and grasslands, as well as agricultural fringe areas. Common prey species for wild felids include: sambar (*Cervus unicolor*), barking deer (*Muntiacus muntjak*), wild pigs (*Sus scrofa*), serow (*Capricornis thar*), goral (*Naemorhedous goral*), Himalayan crestless porcupine (*Hystrix brachyuran)*, langurs (*Trachypithecus spp*), macaques (*Macaca spp*.) and several bird species.

### Ethics Statement

The Ministry of Agriculture and Forests, Royal Government of Bhutan, approved this study as a part of long-term predator monitoring in Royal Manas National Park. The Ugyen Wangchuck Institute for Conservation and Environment (UWICE) conducted this study in collaboration with the University of Montana. UWICE is a governmental research institute with the mandate to conduct studies in the protected areas of Bhutan. Since we did not handle any animals and used non-invasive, remote camera traps, no animal handling, care nor ethics permits were necessary.

### Field Methods

We conducted a camera trapping study in the lower foothills of the Manas administrative range of RMNP. This area was chosen due to high expected wild felid diversity and to minimize logistical and security constraints [33]. We placed pairs of camera traps (Reconyx Inc., Holmen, WI, U.S.A.) in 29 locations within this area from mid-November to mid-February for a total of 91 days. To distribute camera traps, we first created a grid of 2.5 km x 2.5 km cells that covered 162 km^2^, although international and topographic boundaries made some irregular (Fig 1). We then distributed available camera pairs among the 56 total cells, but logistical constraints sometimes prevented camera placement in accordance with the grid, such that three grid cells had two pairs of cameras and one camera pair was placed outside of the grid.

We placed cameras along walking paths and animal trails that showed signs of wildlife use to maximize the probability of photographing target felid species. Cameras were mounted on trees or cut poles and placed two to four meters from the focal movement pathway. We cleared vegetation between the camera and the trail. To minimize elephant damage to cameras, we placed fresh elephant dung on and around cameras. We did not move camera traps during the 91-day sampling period and visited cameras as needed (every two to four weeks) to change batteries and memory cards. We identified individuals based on unique pelage patterns and determined the sex of these individuals as possible from sex-specific cues, such as visible genitalia or the presence of young. We performed this identification visually without the aid of any pattern-recognition software. A single author (TT) verified all leopard identifications to minimize any observer effects.

### Analytical Methods

We estimated the abundance and density of leopards within our study area using a spatial capture-recapture model [14, 21, 23, 34]. We followed the hierarchical model formulation described by Royle *et al*. [35] for a camera-trapping grid. The model relates the observations, *y*_*ijk*_, of individual *i* in trap *j* during sampling interval *k* to the latent distribution of activity centers. We let the observation, *y*_*ijk*_, take the value of one for a capture and zero if not captured to produce a capture history for all individuals in all traps over all sampling intervals. We treated multiple detections of an individual in a particular trap during the same sampling interval as a single capture. Individuals could be captured in multiple traps during a sampling interval. We followed the formulation of the observation process used by Gardner *et al*. [36] and Russell *et al*. [23]. Within this model structure, we allowed the shape of the movement distribution to vary between an exponential and half-normal model (*i*.*e*., 1 ≤ θ ≤ 2, where θ represents the exponent of the kernel from the exponential family of distributions).

We fit a suite of 16 models to the data to assess the impact of varying the duration of sampling intervals and different covariates. We used 4 different sampling interval durations, corresponding to daily (91 sampling occasions), weekly (13 sampling occasions), monthly (three sampling occasions) and quarterly (one sampling occasion) sample periods. For each of these durations, we fit four different covariate combinations: (1) a model where detectability depended only on distance between a trap and the individual’s activity center (Distance), (2) a model with sex as a covariate on baseline detectability (Sex), (3) a model with sex as a covariate on the scale of the movement distribution (σ_sex_), and (4) a model with sex as a covariate on baseline detectability and the scale of the movement distribution (Sex + σ_sex_). We fit these models using an area that buffered the trapping grid by 10 km, yielding a state space of 1,551 km^2^. This buffer incorporates individuals with activity centers outside of the trapping grid, but whose movement range extends into the trapping grid, allowing for a robust estimate of density.

### Bayesian Model Analysis

We used a Bayesian approach to model analysis using data augmentation [14, 37, 38], which has been successfully implemented in a number of recent SCR models [21, 23, 36]. This technique adds a sufficiently large number of all-zero (un-encountered) capture histories to create a dataset of size *M* individuals. We determined the augmentation to be large enough, when the number of augmented individuals did not limit posterior estimates of population size. We chose a uniform prior distribution from [0, *M*] on population size.

We fit our models using Markov chain Monte Carlo (MCMC) methods in R [39], using the SCRbayes package (available at: https://sites.google.com/site/spatialcapturerecapture/scrbayes-r-package; S1 File). We ran models for 20,000 iterations, discarded the first 5,000 iterations as burn-in and further thinned the chain by skipping every other iteration to reduce autocorrelation, leaving 7,500 iterations in our posterior sample. We assessed the convergence of the MCMC samples by examining trace plots and histograms for each parameter. From these converged samples, we computed the mean, median and 95% credibility intervals for the model parameters.

### Model Selection

The flexibility of SCR models to incorporate covariates at different levels allows estimates to include added complexity, but also presents ecologists with potential model selection problems when using Bayesian methods. Estimates and precision may vary among different models creating uncertainty regarding the population status and preventing insight into the mechanisms generating patterns of abundance [12, 23, 40]. These model selection problems can be especially troublesome in an applied context, where managers must make decisions regarding where and how to distribute limited resources.

To assess the relative merit of the different model parameterizations and bridge this methodological gap, we computed the posterior model probabilities for models with daily sampling intervals. Let each model parameterization be represented by M_*l*_ for *l* = 0,…,3, for the data, **y**. From Bayes’s theorem, the relative posterior probabilities among the models in the candidate set are given by

$$\text{Pr}\left({Μ}_{l}|\text{y}\right)=\frac{\text{Pr}\left(\text{y}|{Μ}_{l}\right)\text{Pr}\left({Μ}_{l}\right)}{\underset{l}{\sum }\text{Pr}\left(\text{y}|{Μ}_{l}\right)\text{Pr}\left({Μ}_{l}\right)}$$

Where Pr(**y|**M_*l*_) is the likelihood of the data given the model and Pr(M_*l*_) is the prior probability of a model being correct. For our evaluation, we chose an uninformative prior and set Pr(M_*l*_) = 0.25 for all models. Under this assumption, Bayes’s theorem then reduces to the proportion of the total likelihood attributed to a particular model. Gelfand and Dey [41] proposed an approximation to this quantity using the posterior distribution of the model parameters, such that

$$\text{Pr}\left(\text{y}|{Μ}_{l}\right)\approx {\left(\frac{1}{c}\overset{c}{\underset{i=1}{\sum }}\frac{f\left(\omega_{l}^{\left(i\right)}\right)}{\text{Pr}\left(\text{y}|\omega_{l}^{\left(i\right)},{Μ}_{l}\right)\text{Pr}\left(\omega_{l}^{\left(i\right)}|{Μ}_{l}\right)}\right)}^{-1}$$

Where *f*(·) is a probability density function of the same dimension as ***ω***_***l***_, the parameters in the model, and ***ω***_***l***_^***(i)***^ is a sample from the posterior distribution for a particular iteration of the MCMC, *i* = 1,…, *c*. For our models, *c* took the value of 7,500, the number of posterior samples. The denominator is the likelihood multiplied by the density at the value of the parameters for any given iteration of the chain. We chose *f*(·)~*t*_*7500*_(${\bar{\omega }}_{l}^{i}$, ∑), a multivariate t-distribution with 7,500 degrees of freedom, centered at the mean of the posterior samples and with a scale matrix, ∑, the correlation matrix of the samples. The Gelfand and Dey [41] approximation compares favorably to alternative approximations of the marginal likelihood [42, 43]. Substituting these marginal likelihoods into Bayes’s theorem above gives the relative probability of each model given the data.

We assessed the support of the additional sex-specific parameters for detectability and scale of the movement distribution using Bayes factors [44, 45]. To compute the Bayes factor between the distance-only model, M_*0*_, and each of the sex-specific models, M_*l*_ (*l* = 1, 2, 3), we used

$$B_{l0}=\frac{\text{Pr}\left(\text{y}|{Μ}_{l}\right)}{\text{Pr}\left(\text{y}|{Μ}_{0}\right)}$$

which describes the ratio of the posterior odds to prior odds for M_*l*_ and M_*0*_ [42]. The Bayes factor provides a Bayesian analog to the likelihood ratio test, although Bayes factors differ in two important respects. Bayes factors use the integrated marginal likelihood instead of the maximized likelihood, such that model uncertainty is incorporated into the test statistic, and Bayes factors do not require that hypotheses have nested forms.

### Simulations

We conducted a simulation study to corroborate our findings with respect to the effects of sampling interval on estimated density and precision, and test our model selection techniques. We simulated 89 SCR datasets for a population of size N = 250 (150 female, 100 male) distributed over an 11 x 11 unit continuous statespace for a density of 1.98 individuals/unit^2^. Our simulations exposed these individuals to an 8 x 8 trap grid centered within the statespace for 90 (daily) sampling occasions. We generated capture histories using a Binomial observation model with a half-normal hazard rate detection function and sex-specific baseline detection probabilities, i.e. the Sex model. To parameterize the data generation, we used estimates from models fit to our field data. We set σ = 1 unit, λ_0, female_ = 0.05 and β_sex_ = -1.61. For each simulation, we then subsampled the data to have 1 (quarterly), 3 (monthly) and 13 (weekly) sampling occasions. Finally, we fit the Distance, Sex, σ_sex_, and Sex + σ_sex_ models to all temporal resolutions of each simulated dataset. To fit these models, we discretized the statespace into an 11.5 x 11.5 grid of points and fixed θ = 1 (a half-normal hazard rate detection function). We ran all models with 5,000 MCMC iterations, discarding the first 1,000 iterations as burn-in. We use these results to assess the accuracy and precision of model estimates and the fidelity of our Bayesian model selection methods.

## Results

### Camera Trapping

We captured 22 individual common leopards a total of 82 times between 17 November 2010 and 15 February 2011 in 2,639 camera-trapping nights. The median number of captures per individual was 2 (range: 1–16 captures) and individuals were captured in between one and eight different traps in daily sampling opportunities. We identified six of these individuals as males and the remainder (16 leopards) as female based on photographic evidence of genetalia and/or offspring. We considered camera stations to have operated continuously for the duration of the study, since both cameras at a trap location never malfunctioned simultaneously.

### Model Results

**Fig 2:**
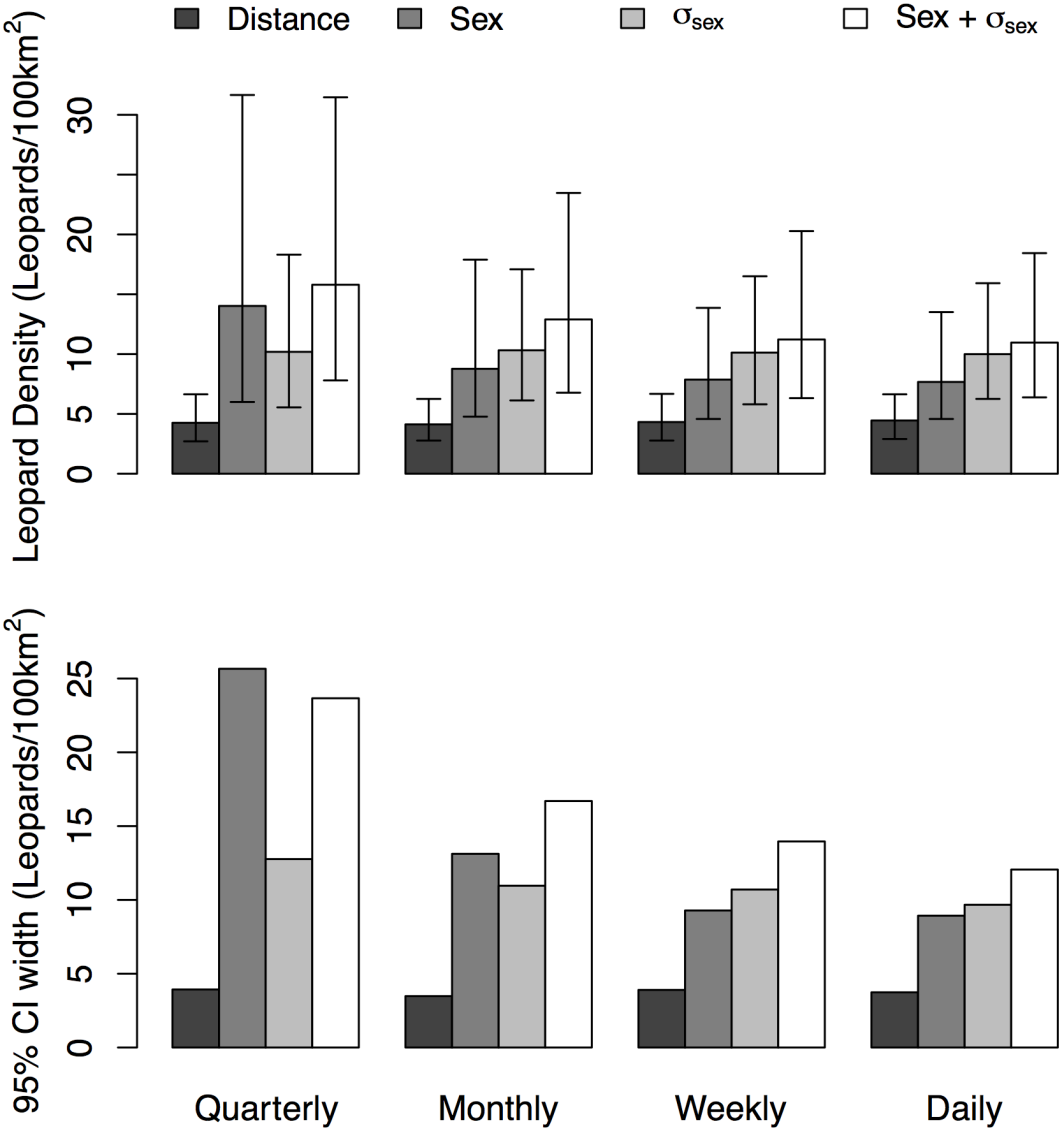
Variation in Median Leopard Density Estimates and 95% Credibility Interval Width for Different Sampling Intervals. Effects of changing sample period duration on median estimated densities (in leopards/100 km^2^; A) and 95% credibility interval width (in leopards/100 km^2^; B) of common leopards in Royal Manas National Park, Bhutan during winter 2010–2011 for 16 spatial capture-recapture models. The 16 models include all possible combinations of the four sampling periods—Quarterly, Monthly, Weekly and Daily—and four model specifications—Distance (dark gray), Sex (medium gray), σ_sex_ (light gray) and Sex + σ_sex_ (white). Error bars represent 95% credibility intervals.

We fit 16 models to the camera captures of common leopard we obtained from sampling. The density estimates varied from a minimum of 4.1 leopards/100 km^2^ in the Distance-only model with monthly sampling periods to 15.8 leopards/100 km^2^ in the sex + σ_sex_ model with a single, quarterly sampling interval (Table 1). For all sampling interval lengths, the Distance model estimated a lower density of animals than any of the models with sex-specific parameters, such that the Distance model median did not fall within the 95% credibility intervals (CI) of the estimates from the other models. The models with sex-specific parameters provided comparable density estimates and none differed at the 95% credibility level (Table 1; Fig 2). Sampling interval length affected the density estimates of models including a sex-specific baseline detection probability, as the density estimates of the Sex and Sex + σ_sex_ model declined with shorter sampling intervals (Table 1; Fig 2). These trends, however, were not significant at the 95% credibility level. The precision of the density estimates also varied with sampling interval length. Shorter sampling intervals typically yielded narrower 95% CIs for all models, especially the Sex and Sex + σ_sex_ models, although we note that the Distance model with a weekly sampling interval did not follow this pattern (Table 1; Fig 2). Sampling interval length also produced a distinct pattern in the baseline detection probability (λ_0_) and sex-specific covariate on this parameter (β_sex_). Shorter sampling intervals had lower baseline detection probabilities (for both sexes, in sex-specific models) (S2 Table). This result matches the intuitive expectation that the detection probability declines as the interval length becomes shorter.

**Table 1:** Common Leopard Population Density Estimates, 95% Credibility Intervals and Standardized 95% Credibility Interval Width for All Sampling Intervals and Model Parameterizations.

Model	Quarterly			Monthly			Weekly			Daily		
	Median	95% CI	SW^a^	Median	95% CI	SW^a^	Median	95% CI	SW^a^	Median	95% CI	SW^a^
Distance	4.3	2.71–6.64	0.92	4.1	2.77–6.25	0.84	4.3	2.77–6.68	0.90	4.5	2.90–6.64	0.84
Sex	14.0	6.00–31.66	1.83	8.8	4.77–17.89	1.50	7.9	4.58–13.86	1.18	7.7	4.58–13.51	1.16
σ_sex_	10.2	5.55–18.31	1.25	10.3	6.13–17.09	1.06	10.1	5.80–16.51	1.06	10.0	6.25–15.93	0.97
Sex + σ_sex_	15.8	7.80–31.46	1.50	12.9	6.77–23.47	1.30	11.2	6.32–20.28	1.24	11.0	6.38–18.44	1.10

Population density estimates (leopards/100 km^2^) of common leopards in a portion of Royal Manas National Park, Bhutan during 2010–2011 from MCMC samples of spatial capture-recapture models. Estimates are reported as medians, 95% credibility intervals (95% CI) and standardized widths of the 95% credibility intervals (SW) from models with covariates for distance, Sex, σ_sex_, and Sex + σ_sex_ with four subdivisions of the data into quarterly, monthly, weekly and daily sampling intervals.

^a^ Standardized widths of 95% credibility intervals calculated by taking the difference of upper and lower 95% credibility bounds and dividing by the median.

In models with a daily sampling interval, the σ_sex_ model received 0.971 of the posterior model probability among the four candidate model parameterizations. The Bayes factor comparison to the distance model showed strong support for this model, as it was over 33 times as likely as the distance model and had positive values of log_10_ *B*_l0_ and 2 ln *B*_l0_ (Table 2). This model estimated a median density of 10.0 leopards/100 km^2^ (95% CI of 6.25–15.93 leopards/100 km^2^) (Fig 1), which corresponds to an abundance of 155 leopards (95% CI: 97–247 leopards) within the statespace.

**Table 2:** Bayesian Model Selection Results for Daily Sampling Interval Models for Common Leopards.

Model	Pr(Model|data)	*B*_*10*_	log_10_ *B*_*l0*_	2 ln *B*_*l0*_
Distance	0.029	1	0	0
Sex	< 0.001	< 0.01	-5.44	-25.03
σ_sex_	0.971	33.35	1.52	7.01
Sex + σ_sex_	< 0.001	< 0.01	-4.72	-21.75

Bayesian model selection results for spatially explicit capture-recapture models with daily sampling intervals for common leopards in Royal Manas National Park, Bhutan during 2010–2011. Pr(Model|data) gives the posterior probability of a model given the data among the candidate models. The Bayes factor, *B*_l0_, provides a Bayesian analog to the likelihood ratio of a model compared to the null, distance model, where values greater than one indicate support for the alternative model. log_10_ *B*_l0_ and 2 ln *B*_l0_ describe different transformations of the Bayes factor to the log_10_ scale as suggested by Jeffreys (1961) and twice the natural logarithmic scale as suggested by Kass & Raftery (1995), respectively. These transformations correspond to the familiar log-odds scale of logistic regression and deviance scale of many information criteria, respectively. For both transformations, values greater than zero favor the alternative hypothesis.

### Simulations

The simulated datasets represented capture histories for a median of 223 unique individuals (95% CI: 212.0–232.6) recorded a median of 2479 times (95% CI: 2266.0–2752.2) over 90 sampling occasions, 2325 times (95% CI: 2111.8–2569.0) over 13 sampling occasions, 1864 times (95% CI: 1703.2–2053.0) over 3 sampling occasions and 1255 times (95% CI: 1142.8–1360.8) over a single sampling interval. On average, capture events recorded 144 female (95% CI: 138.2–148.0) and 79 male individuals (95% CI: 70.4–86.0).

The median of median density estimates across simulated datasets for each model and sample interval length varied from a minimum of 1.84 individuals/unit^2^ with the Distance model, using either daily or weekly sampling periods, to a maximum of 2.35 individuals/unit^2^ with the Sex + σ_sex_ model with a quarterly sampling period (Table 3). The Distance model produced significantly lower density estimates than models that incorporated sex-specific parameters, but density estimates from models that included sex-specific parameters did not differ at the 95% credibility level (Table 3). This pattern reflects a negative bias of estimates from the Distance model and a positive bias of estimates from models with sex-specific detection parameters.

**Table 3:** Simulated Population Density Estimates, 95% Credibility Intervals and Standardized 95% Credibility Interval Width for All Sampling Intervals and Model Parameterizations.

Model	Quarterly			Monthly			Weekly			Daily		
	Median	95% CI	SW^a^	Median	95% CI	SW^a^	Median	95% CI	SW^a^	Median	95% CI	SW^a^
Distance	1.87	1.792–1.951	0.086	1.85	1.784–1.936	0.081	1.84	1.777–1.921	0.079	1.84	1.777–1.921	0.078
Sex	2.31	2.140–2.526	0.166	2.29	2.125–2.488	0.157	2.28	2.117–2.473	0.154	2.28	2.117–2.465	0.156
σ_sex_	2.20	2.049–2.374	0.139	2.23	2.087–2.397	0.141	2.25	2.095–2.420	0.140	2.25	2.102–2.420	0.142
Sex + σ_sex_	2.35	2.163–2.563	0.171	2.32	2.140–2.533	0.167	2.32	2.147–2.526	0.163	2.32	2.140–2.526	0.166

Population density estimates (individuals/unit^2^) from Bayesian spatial capture-recapture models for simulated data with a true density of 1.98 individuals/unit^2^. Estimates are reported as the median of each simulation’s median, 95% credibility intervals (95% CI) and standardized widths of the 95% credibility intervals (SW) from models with covariates for distance, Sex, σ_sex_, and Sex + σ_sex_ with four subdivisions of the data into quarterly, monthly, weekly and daily sampling intervals.

^a^ Standardized widths of 95% credibility intervals calculated by taking the difference of upper and lower 95% credibility bounds and dividing by the median.

Precision of the density estimates depended upon sampling interval length. The Distance, Sex and Sex + σ_sex_ models showed improved precision when moving from quarterly sampling interval to finer temporal resolutions (monthly, weekly or daily sampling intervals) (Table 3). Precision did not differ substantially or predictably among monthly, weekly or daily sampling intervals (Table 3).

The Sex model received the greatest support across all simulations for all sampling intervals. The Bayes factors model selection process identified the Sex model as having the highest posterior probability at a minimum of 60.7% of simulations with a weekly sampling interval and as much as 71.9% of simulations with quarterly sampling intervals (Table 4). As the most frequent top model choice, the Sex model had the highest median posterior probability estimate in all sampling intervals, receiving a minimum of 94% median posterior model probability with weekly sampling intervals (Table 4). These posterior probabilities varied significantly across simulations for models with sex-specific baseline detection probabilities, as the 95% credibility intervals of the Sex and Sex + σ_sex_ models ranged from 0 to 1. However, the Distance and σ_sex_ model received relatively little support across all simulations and sampling intervals with the σ_sex_ model identified as the top model in 2.2% of simulation replicates with quarterly sampling intervals.

**Table 4:** Bayesian Model Selection Results for Daily Sampling Interval Models Fit to Simulated Data.

Model	Quarterly		Monthly		Weekly		Daily	
	Top Model %	Pr(Model|data)	Top Model %	Pr(Model|data)	Top Model %	Pr(Model|data)	Top Model %	Pr(Model|data)
Distance	0	0 (0,0)	0	0 (0,0)	0	0 (0,0)	0	0 (0,0)
Sex	71.9	0.996 (0, 1.000)	66.3	0.998 (0, 1.000)	60.7	0.944 (0, 1.000)	64.0	0.997 (0, 1.000)
σ_sex_	2.2	0 (0, 0.2778)	0	0 (0,0)	0	0 (0,0)	0	0 (0,0)
Sex + σ_sex_	25.8	0.002 (0, 1.000)	33.7	0.002 (0, 1.000)	39.3	0.056 (0, 1.000)	36.0	0.003 (0, 1.000)

Summary of Bayesian model selection results for all simulated datasets with daily, weekly, monthly and quarterly sampling intervals, and Distance, Sex, σ_sex_ and Sex + σ_sex_ models. Top Model % gives the percent of simulations that identified a particular model as the having the highest posterior probability. Pr(Model|data) gives the median posterior probability estimate of a model given the data within the model set with 95% credibility interval of this probability in parentheses. The Sex model represents the true data generating process for all simulations.

## Discussion

We provide two advances in the implementation and use of SCR models through a simulation study and our analysis of the common leopard population in the lower foothills RMNP, Bhutan. First, our investigation of different sample interval lengths provides some guidance to reduce the variability of SCR population estimates, particularly for difficult to detect species, like large carnivores. Second, we apply Bayes factors in a novel context to perform SCR model selection. These results suggest new insights and avenues for future development of these methods within population ecology.

### Sample Interval Length and SCR Estimates

Sample interval length affected the precision of density estimates in our SCR analysis of the leopard data and our simulation study. For the common leopard, we found that analyzing our data with daily sample intervals provided the best precision for all models, especially those with covariates (Table 1; Fig 2). The advantages of daily sampling intervals for model precision did not hold in our simulations, where the principle improvement in precision occurred when moving from a quarterly sampling interval to any finer temporal resolution (Table 3). We believe these divergent results largely depend upon the size of the leopard versus the simulated datasets. Even at our finest temporal resolution (daily sampling intervals), we recorded only 82 leopard captures, a relatively sparse dataset by any measure. In contrast, our simulated data included 1255 capture events (95% CI: 1142.8–1360.8) at the coarsest, quarterly scale. Despite these relatively rich datasets, we still observed improved precision with shorter sampling intervals that increased the number of unique capture events in our simulation. However, we note that these gains in precision for our simulations were of smaller magnitude than those observed in the leopard models. The differences in the size of the leopard and simulated datasets likely occurred due to a higher density of individuals and the larger, more regular trapping grid of our simulations [18–20], even though we used estimates from our leopard analysis to parameterize the simulations. Thus, the impact of sampling interval length may have an asymptotic relationship with model precision that further depends upon the size of the gathered dataset and by proxy the study design.

These results suggest some guidelines for the implementation of SCR models. For camera trapping surveys of elusive species, where samples have accurate time signatures and limited captures take place, we recommend using daily (or finer, if recapture rates are high) subdivisions of the data [26, 46]. While these short sample intervals do come with added computation time in both likelihood-based and Bayesian analysis, this specificity maximizes the potential number of recaptures of individuals and allows for better estimation of the movement distributions central to the SCR framework. Of course, some sampling techniques, such as hair snares or scat surveys, may not allow such temporal specificity of samples [23, 36]. For these alternative methods, our results demonstrate substantive gains in precision when moving from quarterly sample intervals to monthly or weekly intervals, especially with high capture rates as in our simulations (Tables 1 and 3; Fig 2). Taken together, our study clearly cautions against highly collapsed sampling intervals in SCR studies.

### Bayesian Model Selection

Our simulation study largely validated our Bayes factor model selection methods. The true data generating process in our simulations incorporated a sex-specific baseline detection probability and models that incorporated this sex-specific baseline detection probability received decisive support in almost all simulations (Table 4). The Sex model (with only sex-specific baseline detection probabilities) received the strongest and most frequent support, while the Sex + σ_sex_ model (with an additional parameter for sex-specific movement distributions) represented a clear runner-up across the four different sampling intervals. Moreover, our selection methods more frequently identified the Sex + σ_sex_ model as most probable with weekly, and to a lesser extent monthly and daily sampling intervals (Table 4). While these sampling intervals produced datasets rich enough to demonstrate improved precision (Table 3), they may also have contained enough information to support additional, extraneous model complexity and over-fit the available data. We find support for this view in the overlapping confidence intervals for the σ_male_ and σ_female_ parameters in this model (S2 Table). The σ_sex_ component of the Sex + σ_sex_ model did not capture true sex-specific differences, but rather accounted for small, happenstance variation in some datasets [47]. Thus, our Bayes factors model selection may show some bias towards more complex models with increasing sample size, but these selection errors appear identifiable from examining model estimates.

We may have further improved model precision and reduced the observed bias of the density estimates by refining our simulation approach. Across all sampling interval lengths in our simulations, we observed a negative bias in the median densities estimated by the Distance model and a positive bias in the sex-specific covariate models. We feel that these inaccuracies in the model estimates, particularly the Sex model, which generated the data, likely arise from using a statespace that did not adequately capture the area that contains the activity centers of all detectable individuals [35, 48]. Thus, the model does not account for individuals at the edge of effective sampling area of the trapping array [48]. We find support for this view in the generally negative bias of the σ parameter estimates, especially for more abundant females in the σ_sex_ model (S2 Table). This spurious model parameter likely reduced the bias of the density estimate by accounting for the diminished range for individual movements at the edge of the statespace. Furthermore, this pattern may have contributed to the observed support for the Sex + σ_sex_ model. More generally, this observation raises larger questions about the sensitivity of SCR model results to the definition of the statespace.

Having verified our implementation of the Bayes factors approximation in our simulations, we interpret the model selection results from models of common leopards in RMNP. Among models fit with data from the most precise (daily) sampling interval, median density estimates varied from 4.5 in the Distance model to 11.0 in the Sex + σ_sex_ model (Table 1). Thus, model selection represents a non-trivial matter. On the basis of our Bayes factors approach, we selected the σ_sex_ model, which allows the size of male and female movement ranges to vary. This model received an overwhelming proportion of the posterior model probability (0.971) and was over 33 times more likely than the null, Distance-only model (Table 2). We estimated male movement ranges to be nearly twice the size of female movement ranges (Table 5), which is supported by our recapture of males over a wider range than females. The relatively small movement ranges of females led the model to estimate a relatively large proportion of undetected females, as shown by the strong female bias in sex ratio, ψ_sex_ (Table 5).

**Table 5:** Parameter Estimates and 95% Credibility Intervals from Spatial Capture-Recapture Models with Daily Sampling Intervals for Common Leopards.

Model	λ_0_	β_sex_	σ_male_	σ_female_	ψ_sex_	θ
Distance	0.02 (0.014, 0.041)	0 (0, 0)	2.2 (1.55, 3.05)	2.2 (1.55, 3.05)	0.27 (0.107, 0.520)	0.81 (0.544, 0.988)
Sex	0.01 (0.003, 0.016)	1.59 (0.799, 2.401)	2.0 (1.47, 2.78)	2.0 (1.47, 2.78)	0.17 (0.060, 0.363)	0.82 (0.550, 0.990)
σ_sex_	0.03 (0.016, 0.047)	0 (0, 0)	2.2 (1.56, 3.10)	1.2 (0.96, 1.55)	0.10 (0.036, 0.249)	0.74 (0.510, 0.970)
Sex + σ_sex_	0.01 (0.006, 0.027)	0.99 (0.196, 1.783)	2.2 (1.50, 3.03)	1.4 (1.06, 1.98)	0.10 (0.034, 0.245)	0.78 (0.520, 0.990)

Median parameter estimates with 95% credibility intervals in parentheses from spatial capture-recapture models of common leopards in Royal Manas National Park during 2010–2011 with daily sampling intervals. λ_0_ gives the baseline capture probability at an individual’s activity center per camera station per day. β_sex_ denotes the effect of sex on detection probability on the log scale. The σ parameters describe the scale of an individual’s movement distribution in km, which varies by sex in some models. ψ_sex_ estimates the proportion of the population that is male. θ represents the shape parameter of the individual’s movement distribution, where 0.5 is exponential and 1.0 is Gaussian.

These ecological covariates may entail some cost to the precision of the density estimate. In our simulation and application to the leopard system, sex-specific models, including our top models, had wider 95% CIs than those of the Distance model. For the leopard analysis, we may have improved the precision of the model by arranging our traps in clusters to provide more spatial recaptures of females, as has been shown in simulations and other systems [18–20]. To the contrary, the relative precision of male and female movement ranges indicates that our sampling may have provided a sufficient number of spatial recaptures to adequately estimate this parameter for females. The size of our trapping grid was approximately 2×σ_female_, which falls within recommendations for trap spacing relative to movement distributions from simulations [18, 19]. Overall, this uncertainty underscores the need to maximize both the number of individuals captured and the locations at which they are recaptured in spatial capture-recapture studies [40, 49].

More broadly, the approximation to Bayes factors that we adopt represent only one approach to Bayesian model selection. Hooten and Hobbs [50] provide a timely review of this emerging topic in ecological research, although the appropriateness of the available techniques for SCR models varies. They outline two principle approaches to the problem of Bayesian model selection in the literature. The first class of methods attempts to minimize out-of-sample predictive error by applying some penalty to a measure of model goodness-of-fit. AIC represents the most frequently used method of this type in maximum likelihood analyses. Bayesian analogs to this approach include the deviance information criterion (DIC) [51] and Watanabe-Akaike information criterion (WAIC) [52], however the assumptions of these techniques seem a poor fit for the nature of SCR analyses. Related methods, such as extensions of the WAIC measure [53, 54] or posterior predictive loss [55, 56], may be better suited to SCR models, but come with their own computational challenges [50, 53]. In contrast to these predictive measures of model performance, Bayesian model weighting methods, such as Bayes factors, quantify uncertainty within a set of models given the available data. Promising alternatives to Bayes factors in this wider class of selection techniques include Bernoulli indicator variables (e.g., [57, 58]) or reversible-jump MCMC (RJMCMC) [59], although the former method shows a strong sensitivity to prior distributions that require proper tuning [38, 58] and the latter approach can entail significant technical challenges ([50], but see [60]). Regardless of the chosen model selection tool, researchers should attempt to explore how and under what circumstances each candidate model fails to better understand the underlying ecological process [47, 61].

### Ecological Implications

The support for sex-specific parameters in our leopard example highlights the need to consider relevant behavioral variation in estimating abundance and/or density for large carnivores. Previous leopard radio tracking [62, 63] and camera trapping [40] studies have found variation in home or movement range sizes between males and females. The significant difference between our top model and the distance model shows that failing to consider this factor would have led to an underestimate of leopard density in our study area. Similarly, Gray and Prum [40] found support for sex-specific differences in baseline detection probability, suggesting these differences may occur because males use trails more than females. We found some evidence for sex-specific differences in baseline detectability, since β_sex_ had 95% CIs that did not overlap zero (Table 2). Although alternative approaches to model selection would have included this term in the top model [23], these models had extremely low posterior probabilities in our Bayes factor analysis (<0.001). On the basis of these low posterior probabilities, we rejected these models in favor of the σ_sex_ model, although we note that the point estimates between these models did not differ significantly. In our study, differences in detectability appear to have occurred because males had larger movement ranges than females, not because of inherent differences in baseline detection probability (*e*.*g*. from trail use patterns). Furthermore, our model selection techniques gave additional insight into leopard behavior in our system and improved our density estimate.

A comparison of our estimate to other study areas from the Indian sub-continent demonstrates the importance of considering animal ecology in SCR models [49, 64, 65]. Our estimate of 10.0 leopards/100 km^2^ (95% CI: 6.25–15.93 leopards/100 km^2^) was within the reported range of leopard densities, from 1.0–28.9 leopards/100 km^2^ [65], across their geographic distribution. In the adjoining Indian Manas National Park, Borah *et al*. [49] reported a SCR-based estimate of 3.4 ± 0.82 SE leopards/100 km^2^, similar to our estimate for the Distance model of 4.5 leopards/100 km^2^ (95% CI: 2.90–6.64). However, they did not consider sex-specific covariates, which we found to increase density in our study. The Bayes factor model selection approach demonstrated strong support for sex-specific movement distributions suggesting the SCR-based estimate of Borah *et al*. [49] may be biased low. More broadly, SCR methods in combination with Bayes factors may help resolve the discrepancies that commonly occur in estimates of large carnivore density [12, 23, 65].

Looking towards the future, we see a great deal of promise in SCR models incorporating auxiliary ecological information. Our estimate may have been further improved by incorporating a metric of habitat quality [66], although we did not have any such indices available for our study area. In addition to habitat, leopard density may further depend on the community of sympatric large carnivores. Harihar *et al*. [64] found that leopard density declined from a median estimate of 9.09 leopards/100 km^2^ (95% CI: 4.54–17.98) to 1.44 leopards/100 km^2^ (95% CI: 0.41–6.61), corresponding to an increase in tiger density across years. These contrasting changes in density across an entire study area raise the possibility that conspecific interference or avoidance may affect patterns of abundance or movement within a study at finer spatial and/or temporal scales. These interactions could be integrated into SCR analyses by introducing the presence or abundance of other species as spatial covariates or through multi-species models. More broadly, SCR methods have been integrated with concepts of resource selection and ecological distance to provide inference on factors affecting habitat use and animal movements [22, 67]. These sorts of analyses expand the scope of our understanding, beyond simply density or abundance, of large carnivores, such as leopards.

## Conclusions

Many large carnivores, including leopards, have a broad geographic distribution, but increasingly face threats from habitat disturbance, poaching, inadequate prey resources and human-wildlife conflicts. Despite the many risks facing large carnivores, the status of populations across species’ ranges remains poorly understood. SCR methods hold a great deal of promise to help fill this critical gap of knowledge for large carnivores by explicitly incorporating spatial heterogeneity in detections into our estimates of abundance. These methods yield the greatest precision when combined with a high temporal resolution of sampling (daily intervals), although more modest weekly or monthly sampling intervals can be informative, particularly with high capture rates. Moreover, we implement and validate a Bayesian model selection method for use with these models. These methodological advancements may contribute to population assessments, help prioritize conservation efforts and improve understanding of large carnivore ecology as it relates to population biology. These steps towards a more holistic view of conservation may pay dividends for large carnivores and threatened species globally.

## Supporting Information

S1 File R Code to Implement Spatial Capture Recapture Analysis with Bayes Factors Model Selection. R code to implement spatial capture recapture models with a novel approach to model selection using the SCRbayes package. This script reads the data for the camera trap locations and leopard capture histories available through the Dryad Database (http://dx.doi.org/10.5061/dryad.mr1pt). This data is used to fit four spatial capture-recapture models with the SCRbayes package for R (package available at: https://sites.google.com/site/spatialcapturerecapture/scrbayes-r-package). Finally, we conduct model selection with Bayes factors. Users should consult the package help files and additional resources for further information.
(DOCX)

S2 File Internal R Code to Conduct Bayes Factors Model Selection. This function within the SCRbayes package will operate on standard model output from SCRj.fn to calculate posterior marginal likelihoods and Bayes factors from spatial capture-recapture models implemented in the SCRbayes package. The SCRbayes package can be accessed at: https://sites.google.com/site/spatialcapturerecapture/scrbayes-r-package. This code comes as a function (SCR.bf) within the package. Users should consult the package help files and additional resources for further information.
(DOCX)

S1 Table Parameter Estimates and 95% Credibility Intervals from All Spatial Capture-Recapture Models for Common Leopards. Median parameter estimates with 95% credibility intervals in parentheses from spatially explicit capture-recapture models of common leopards in Royal Manas National Park during 2010–2011 for all 16 combinations of covariates and data. λ_0_ gives the baseline capture probability at an individual’s activity center per sample interval per camera station. β_sex_ denotes the effect of sex on detection probability on the log scale. The σ parameters describe the scale of an individual’s movement distribution in km, which varies by sex in some models. ψ_sex_ estimates the proportion of the population that is male. θ represents the shape parameter of the individual’s movement distribution, where 0.5 is exponential and 1.0 is Gaussian.
(DOCX)

S2 Table Median Parameter Estimates and 95% Credibility Intervals from All Spatial Capture-Recapture Models from Simulations. Median of median model parameter estimates with median 95% credibility intervals in parentheses from spatially explicit capture-recapture models fit to all simulated datasets for all 16 combinations of covariates and data. λ_0_ gives the baseline capture probability at an individual’s activity center per sample interval per camera station. β_sex_ denotes the effect of sex on detection probability on the log scale. The σ parameters describe the scale of an individual’s movement distribution in units, which varies by sex in some models. ψ_sex_ estimates the proportion of the population that is male. True values used to generate the simulated were: λ_0_ = 0.05 (with daily sampling intervals), β_sex_ = -1.61, σ_male_ = σ_female_ = 1.0, ψ_sex_ = 0.4.
(DOCX)
