# Supplementary material for: Examining Temporal Sample Scale and Model Choice with Spatial Capture-Recapture Models in the Common Leopard Panthera pardus
Source: PLoS One. 2015 Nov 4;10(11):e0140757. doi: 10.1371/journal.pone.0140757 (PMC4633112; doi:10.1371/journal.pone.0140757)
Supplement: S1 File — R code to implement spatial capture recapture models with a novel approach to model selection using the SCRbayes package. This script reads the data for the camera trap locations and leopard capture histories available through the Dryad Database (http://dx.doi.org/10.5061/dryad.mr1pt). This data is used to fit four spatial capture-recapture models with the SCRbayes package for R (package available at: https://sites.google.com/site/spatialcapturerecapture/scrbayes-r-package). Finally, we conduct model selection with Bayes factors. Users should consult the package help files and additional resources for further information. (DOCX) [file pone.0140757.s001.docx]

library(chron)

library(SCRbayes)

#Create trapping grid data.frame with ids, coordinates, and open/closed status for each sampling occassion - assummed all open

traps = read.csv("Leopard Camera Traps (for dryad).csv", header=T)

traps<-cbind(traps, V4=matrix(1,nrow=nrow(traps),ncol=91))

#Create state space, entire study area considered habitat

ss.animal<-make.ss(traps, 11250,1000)$ss.animal

ss.animal$habitat = 1

#Read in capture data

captures = read.csv("Leopard Camera Trapping Data (for Dryad).csv",header=T)

# Format date field

captures$Date = chron(as.character(captures$Date),format=c(dates = "d-mon-y"), origin.=c(month=11, day=16,year=2010))

# Make into numeric sampling occasions

captures$SO = as.numeric(captures$Date)

# Order by individual id

captures = captures[order(captures$AnimalID),]

# Re-format and order data for SCRj.fn as EDF

scrCaptures = data.frame(session=rep(1,nrow(captures)), individual = as.numeric(as.factor(captures$AnimalID)), occasion=captures$SO, trapid=captures$TrapID)

scrCaptures = scrCaptures[order(scrCaptures$individual),]

# Format the sex field 0 = F, 1 = M

Xsex<-(as.numeric(captures$Sex)-1)[-which(duplicated(scrCaptures$individual))]

# pass all necessary data to scrData in prep for model fitting

scrobj<- scrData(traps=traps, captures = scrCaptures, statespace = ss.animal, Xsex = Xsex)

#Define and run models of interest.

model1 <-SCRj.fn(ni=5000, burn=1000, skip=1,nz=200,scrobj=scrobj, Msigma=1, Mb=0, Msex=0, Msexsigma=0, Meff=0, Mss=0, Mtel=0, theta=NA, coord.scale=2500,area.per.pixel=1,thinstatespace=1, maxNN=20, dumprate=1000)

model2 <-SCRj.fn(ni=5000, burn=1000, skip=1,nz=200,scrobj=scrobj, Msigma=1, Mb=0, Msex=1, Msexsigma=0, Meff=0, Mss=0, Mtel=0, theta=NA, coord.scale=2500,area.per.pixel=1,thinstatespace=1, maxNN=20, dumprate=1000)

model3 <-SCRj.fn(ni=5000, burn=1000, skip=1,nz=200,scrobj=scrobj, Msigma=1, Mb=0, Msex=0, Msexsigma=1, Meff=0, Mss=0, Mtel=0, theta=NA, coord.scale=2500,area.per.pixel=1,thinstatespace=1, maxNN=20, dumprate=1000)

model4 <-SCRj.fn(ni=5000, burn=1000, skip=1,nz=200,scrobj=scrobj, Msigma=1, Mb=0, Msex=1, Msexsigma=1, Meff=0, Mss=0, Mtel=0, theta=NA, coord.scale=2500,area.per.pixel=1,thinstatespace=1, maxNN=20, dumprate=1000)

# Place all models in named list

model.list = list('Distance'=model1, 'Sex'= model2, 'SexSigma'=model3, 'SexSexSigma'=model4)

# Conduct Bayes factor model comparison.

BF.table = SCR.bf(model.list, refmodel=1)
