# Supplementary material for: Examining Temporal Sample Scale and Model Choice with Spatial Capture-Recapture Models in the Common Leopard Panthera pardus
Source: PLoS One. 2015 Nov 4;10(11):e0140757. doi: 10.1371/journal.pone.0140757 (PMC4633112; doi:10.1371/journal.pone.0140757)
Supplement: S2 File — This function within the SCRbayes package will operate on standard model output from SCRj.fn to calculate posterior marginal likelihoods and Bayes factors from spatial capture-recapture models implemented in the SCRbayes package. The SCRbayes package can be accessed at: https://sites.google.com/site/spatialcapturerecapture/scrbayes-r-package. This code comes as a function (SCR.bf) within the package. Users should consult the package help files and additional resources for further information. (DOCX) [file pone.0140757.s002.docx]

SCR.bf = function(modelsin, refmodel=NULL){

require(mvtnorm)

nmodels = length(modelsin)

model.lik <- rep(NA, nmodels)

n.nolik <- list()

LLsamples <-LLs<- matrix(nrow=nrow(modelsin[[1]]$likelihood),ncol=nmodels)

LLsamples <- sapply(modelsin,"[[",'likelihood')[1:nrow(modelsin[[1]]$likelihood),]

maxLL <- max(LLsamples)

LLprime <- LLsamples-maxLL

for (i in 1:nmodels){

model = modelsin[[i]]

model.post = model$mcmchist[,model$parms2report]

model.post = model.post[,colnames(model.post)!="sigma"&colnames(model.post)!="sigma2" &colnames(model.post)!="D"&colnames(model.post)!="lam0"]

post.avg = apply(model.post,2,mean)

post.var = var(model.post)

emp.dens = apply(model.post,2,density)

emp.dens = lapply(emp.dens,function(x){

prob<-x$y/sum(x$y)

return(cbind(x=x$x,prob))})

chain.dens = matrix(NA, nrow=nrow(model.post), ncol=ncol(model.post))

for (c in 1:ncol(model.post)){

chain.dens[,c]=emp.dens[[c]][,2][findInterval(model.post[,c],emp.dens[[c]][,1])]

}

f.samples = dmvt(model.post,delta=post.avg, sigma=cov2cor(post.var), df=nrow(model.post),log=T)

model.lik[i] = (1/nrow(model.post)*sum(exp(f.samples-(LLprime[,i]+log(apply(chain.dens,1,prod))))))^(-1)

n.nolik[[i]] <- c("sum"=sum(model$likelihood[,2]), "mean"=mean(model$likelihood[,2]), "median"=median(model$likelihood[,2]), min = min(model$likelihood[,2]), "max"=max(model$likelihood[,2]))

# maxL[i] = max(model$likelihood)

# avgL[i] = mean(model$likelihood)

# medL[i] = median(model$likelihood)

}

post.prob =(model.lik)/sum((model.lik))

names(model.lik) <- names(modelsin)

n.nolik.df = do.call("rbind", n.nolik)

if(!is.null(refmodel)){

bf = model.lik/model.lik[refmodel]

Jeff.breaks = c(1,3,10,30,100)

Jeff.evid = c("Supports Null", "Barely worth mentioning", "Substantial","Strong","Very Strong","Decisive")

KR.breaks = c(1,3,20,150)

KR.evid = c("Supports Null","Barely worth mentioning", "Positive","Strong","Very Strong")

out.table = data.frame("Model" = names(modelsin), "Post.Prob" = post.prob, "BayesFactor"=bf, "log10BF" = log10(bf), "Two*lnBF" = 2*log(bf), "JeffreysEvidence" = Jeff.evid[findInterval(bf, Jeff.breaks)+1],"KRevidence" = KR.evid[findInterval(bf, KR.breaks)+1])

out.table$JeffreysEvidence = factor(out.table$JeffreysEvidence,levels = c(levels(out.table$JeffreysEvidence),"Null Model"))

out.table$KRevidence = factor(out.table$KRevidence,levels = c(levels(out.table$KRevidence),"Null Model"))

out.table[refmodel,c("JeffreysEvidence","KRevidence")]="Null Model"

rownames(out.table)=NULL

out = list("ModelSelection"=out.table, "LowLikelihoodObservations"=n.nolik.df)

return(out)

} else {

out.table = data.frame("Model" = names(modelsin), "Post.Prob" = post.prob)

rownames(out.table)=NULL

out = list("ModelSelection"=out.table, "LowLikelihoodObservations"=n.nolik.df)

return (out)

}

}
