# Supplementary material for: Examining Temporal Sample Scale and Model Choice with Spatial Capture-Recapture Models in the Common Leopard Panthera pardus
Source: PLoS One. 2015 Nov 4;10(11):e0140757. doi: 10.1371/journal.pone.0140757 (PMC4633112; doi:10.1371/journal.pone.0140757)
Supplement: S1 Table — Median parameter estimates with 95% credibility intervals in parentheses from spatially explicit capture-recapture models of common leopards in Royal Manas National Park during 2010–2011 for all 16 combinations of covariates and data. λ0 gives the baseline capture probability at an individual’s activity center per sample interval per camera station. βsex denotes the effect of sex on detection probability on the log scale. The σ parameters describe the scale of an individual’s movement distribution in km, which varies by sex in some models. ψsex estimates the proportion of the population that is male. θ represents the shape parameter of the individual’s movement distribution, where 0.5 is exponential and 1.0 is Gaussian. (DOCX) [file pone.0140757.s003.docx]

| Model/  Data | λ_0_ | β_sex_ | σ_male_ | σ_female_ | ψ_sex_ | θ |
| --- | --- | --- | --- | --- | --- | --- |
| Distance/  Quarterly | 1.58 (0.693, 3.969) | 0 (0, 0) | 2.2 (1.55, 3.36) | 2.2 (1.55, 3.36) | 0.27 (0.112, 0.5) | 0.75 (0.513, 0.988) |
| Sex/  Quarterly | 0.24 (0.073, 0.934) | 2.86 (1.638, 4.274) | 1.9 (1.34, 2.93) | 1.9 (1.34, 2.93) | 0.08 (0.022, 0.22) | 0.72 (0.512, 0.982) |
| σ_sex_ /  Quarterly | 1.33 (0.645, 3.555) | 0 (0, 0) | 2.6 (1.6, 4.91) | 1.4 (0.99, 1.92) | 0.09 (0.027, 0.254) | 0.77 (0.515, 0.992) |
| Sex + σ_sex_ /  Quarterly | 0.34 (0.088, 1.345) | 2.13 (0.726, 3.832) | 2.5 (1.55, 4.15) | 1.7 (1.1, 2.52) | 0.06 (0.016, 0.162) | 0.83 (0.526, 0.993) |
| Distance/  Monthly | 0.75 (0.405, 1.632) | 0 (0, 0) | 2.0 (1.49, 3.12) | 2.0 (1.49, 3.12) | 0.27 (0.107, 0.51) | 0.71 (0.511, 0.987) |
| Sex/  Monthly | 0.13 (0.051, 0.326) | 2.02 (1.128, 2.978) | 2.2 (1.61, 2.98) | 2.2 (1.61, 2.98) | 0.13 (0.043, 0.308) | 0.84 (0.574, 0.992) |
| σ_sex_ /  Monthly | 0.68 (0.379, 1.373) | 0 (0, 0) | 2.3 (1.6, 3.69) | 1.2 (0.94, 1.65) | 0.09 (0.032, 0.225) | 0.71 (0.514, 0.977) |
| Sex + σ_sex_ /  Monthly | 0.20 (0.077, 0.603) | 1.56 (0.410, 2.655) | 2.4 (1.62, 3.39) | 1.5 (1.11, 2.22) | 0.08 (0.025, 0.206) | 0.81 (0.539, 0.993) |
| Distance/  Weekly | 0.19 (0.11, 0.378) | 0 (0, 0) | 2.0 (1.46, 2.84) | 2.0 (1.46, 2.84) | 0.27 (0.11, 0.507) | 0.70 (0.512, 0.967) |
| Sex/  Weekly | 0.05 (0.022, 0.115) | 1.65 (0.949, 2.476) | 2.0 (1.43, 2.67) | 2.0 (1.43, 2.67) | 0.15 (0.056, 0.352) | 0.77 (0.525, 0.985) |
| σ_sex_ /  Weekly | 0.18 (0.105, 0.344) | 0 (0, 0) | 2.1 (1.53, 3.21) | 1.2 (0.93, 1.58) | 0.10 (0.034, 0.232) | 0.69 (0.511, 0.972) |
| Sex + σ_sex_ /Weekly | 0.05 (0.02, 0.137) | 1.30 (0.447, 2.419) | 2.4 (1.74, 3.33) | 1.6 (1.15, 2.31) | 0.09 (0.031, 0.238) | 0.86 (0.603, 0.994) |
| Distance/  Daily | 0.02 (0.014, 0.041) | 0 (0, 0) | 2.2 (1.55, 3.05) | 2.2 (1.55, 3.05) | 0.27 (0.107, 0.520) | 0.81 (0.544, 0.988) |
| Sex/  Daily | 0.01 (0.003, 0.016) | 1.59 (0.799, 2.401) | 2.0 (1.47, 2.78) | 2.0 (1.47, 2.78) | 0.17 (0.060, 0.363) | 0.82 (0.550, 0.990) |
| σ_sex_ /  Daily | 0.03 (0.016, 0.047) | 0 (0, 0) | 2.2 (1.56, 3.1) | 1.2 (0.96, 1.55) | 0.10 (0.036, 0.249) | 0.74 (0.510, 0.970) |
| Sex + σ_sex_ /  Daily | 0.01 (0.006, 0.027) | 0.99 (0.196, 1.783) | 2.2 (1.5, 3.03) | 1.4 (1.06, 1.98) | 0.10 (0.034, 0.245) | 0.78 (0.520, 0.990) |
