# Supplementary material for: Examining Temporal Sample Scale and Model Choice with Spatial Capture-Recapture Models in the Common Leopard Panthera pardus
Source: PLoS One. 2015 Nov 4;10(11):e0140757. doi: 10.1371/journal.pone.0140757 (PMC4633112; doi:10.1371/journal.pone.0140757)
Supplement: S2 Table — Median of median model parameter estimates with median 95% credibility intervals in parentheses from spatially explicit capture-recapture models fit to all simulated datasets for all 16 combinations of covariates and data. λ0 gives the baseline capture probability at an individual’s activity center per sample interval per camera station. βsex denotes the effect of sex on detection probability on the log scale. The σ parameters describe the scale of an individual’s movement distribution in units, which varies by sex in some models. ψsex estimates the proportion of the population that is male. True values used to generate the simulated were: λ0 = 0.05 (with daily sampling intervals), βsex = -1.61, σmale = σfemale = 1.0, ψsex = 0.4. (DOCX) [file pone.0140757.s004.docx]

| Model/  Data | λ_0_ | β_sex_ | σ_male_ | σ_female_ | ψ_sex_ |
| --- | --- | --- | --- | --- | --- |
| Distance/  Quarterly | 2.51 (2.265, 2.79) | 0 (0, 0) | 1.06 (1.024, 1.097) | 1.06 (1.024, 1.097) | 0.35 (0.293, 0.419) |
| Sex/  Quarterly | 4.52 (3.985, 5.183) | -1.78 (-1.989, -1.555) | 0.99 (0.959, 1.026) | 0.99 (0.959, 1.026) | 0.47 (0.396, 0.54) |
| σ_sex_ /  Quarterly | 2.77 (2.502, 3.092) | 0 (0, 0) | 0.73 (0.695, 0.777) | 1.1 (1.058, 1.136) | 0.44 (0.372, 0.51) |
| Sex + σ_sex_ /  Quarterly | 4.47 (3.926, 5.162) | -1.69 (-2, -1.428) | 0.96 (0.885, 1.042) | 1 (0.96, 1.034) | 0.47 (0.403, 0.546) |
| Distance/  Monthly | 1.06 (0.981, 1.136) | 0 (0, 0) | 1.03 (1.003, 1.061) | 1.03 (1.003, 1.061) | 0.35 (0.292, 0.418) |
| Sex/  Monthly | 1.51 (1.395, 1.631) | -1.72 (-1.899, -1.553) | 0.99 (0.965, 1.018) | 0.99 (0.965, 1.018) | 0.46 (0.393, 0.533) |
| σ_sex_ /  Monthly | 1.1 (1.024, 1.18) | 0 (0, 0) | 0.7 (0.664, 0.731) | 1.07 (1.036, 1.1) | 0.45 (0.382, 0.522) |
| Sex + σ_sex_ /  Monthly | 1.49 (1.377, 1.614) | -1.67 (-1.91, -1.457) | 0.95 (0.882, 1.032) | 0.99 (0.967, 1.025) | 0.47 (0.401, 0.544) |
| Distance/  Weekly | 0.26 (0.248, 0.281) | 0 (0, 0) | 1.02 (0.997, 1.049) | 1.02 (0.997, 1.049) | 0.35 (0.292, 0.418) |
| Sex/  Weekly | 0.35 (0.326, 0.37) | -1.71 (-1.878, -1.546) | 0.99 (0.967, 1.017) | 0.99 (0.967, 1.017) | 0.46 (0.393, 0.532) |
| σ_sex_ /  Weekly | 0.27 (0.253, 0.286) | 0 (0, 0) | 0.68 (0.652, 0.715) | 1.06 (1.03, 1.087) | 0.46 (0.387, 0.527) |
| Sex + σ_sex_ /Weekly | 0.35 (0.324, 0.368) | -1.65 (-1.868, -1.459) | 0.95 (0.883, 1.03) | 0.99 (0.969, 1.023) | 0.47 (0.399, 0.541) |
| Distance/  Daily | 0.04 (0.037, 0.041) | 0 (0, 0) | 1.02 (0.995, 1.046) | 1.02 (0.995, 1.046) | 0.35 (0.293, 0.418) |
| Sex/  Daily | 0.05 (0.047, 0.053) | -1.7 (-1.873, -1.543) | 0.99 (0.968, 1.016) | 0.99 (0.968, 1.016) | 0.46 (0.393, 0.533) |
| σ_sex_ /  Daily | 0.04 (0.037, 0.042) | 0 (0, 0) | 0.68 (0.648, 0.71) | 1.06 (1.03, 1.085) | 0.46 (0.388, 0.528) |
| Sex + σ_sex_ /  Daily | 0.05 (0.047, 0.053) | -1.66 (-1.86, -1.465) | 0.95 (0.885, 1.026) | 0.99 (0.969, 1.021) | 0.47 (0.399, 0.541) |
